# Primary monosymptomatic nocturnal enuresis highlighted in this number of International Brazilian Journal of Urology

**DOI:** 10.1590/S1677-5538.IBJU.2022.06.01

**Published:** 2022-11-01

**Authors:** Luciano A. Favorito

**Affiliations:** 1 Universidade do Estado do Rio de Janeiro Unidade de Pesquisa Urogenital Rio de Janeiro RJ Brasil Unidade de Pesquisa Urogenital - Universidade do Estado do Rio de Janeiro - Uerj, Rio de Janeiro, RJ, Brasil; 2 Hospital Federal da Lagoa Serviço de Urologia Rio de Janeiro RJ Brasil Serviço de Urologia, Hospital Federal da Lagoa, Rio de Janeiro, RJ, Brasil

The November-December number of Int Braz J Urol, the 19th under my supervision, presents original contributions with a lot of interesting papers in different fields: Robotic Surgery, Prostate Cancer, Bladder Outlet Obstruction, BPH, Translational Research, Primary monosymptomatic nocturnal enuresis and UPJO. The papers came from many different countries such as Brazil, USA, Turkey, India, China, Italy, Argentina and Singapure and as usual the editor's comment highlights some of them.

In the present issue we present two important papers about Primary monosymptomatic nocturnal enuresis. Dr. Ribeiro and colleagues from Brazil in page 937 (1) evaluated the hormone profile (brain natrituretic peptide -BNP and anti-diuretic hormone - ADH) and improvement in dry nights in a sample of children before and after surgical treatment of the Upper airway obstruction (UAO) and concluded that surgery for airway obstruction contributed to an increase in BNP without increasing ADH. A total of 85.8% of the children presented partial or complete improvement of their enuresis. Dr. Carvalho and colleagues from Brazil performed in page 944 (2) a interesting study about the relationship between the toilet training process (TT) and Primary monosymptomatic nocturnal enuresis (PMNE) and concluded that the age of onset of TT, acquisition of daytime continence, and the type of equipment were not associated with higher occurrence of PMNE. On the other hand, the Child-Oriented approach was a protective factor for the occurrence of PMNE. The editor in chief would like to highlight the following works too:

Dr. Hu and colleagues from China, presented in page 891 (3) a nice systematic review about the feasibility of 68Ga-PSMA PET/CT in diagnosing primary prostate cancer and concluded that compared with conventional imaging examinations, 68Ga-PSMA PET/CT had higher sensitivity and specificity in detecting primary prostate cancer. At present, most of the studies that used 68Ga-PSMA PET/CT for detecting prostate cancer are retrospective studies. Based on its advantage of high detection rate, the use of 68Ga- PSMA PET/CT in the detection of primary prostate cancer is worthy of promotion.

Dr. Gauhar and colleagues from Singapure and Brazil performed in page 903 (4) a interesting systematic review about the perioperative outcomes, complications, and survival in studies comparing ureteral stent and percutaneous nephrostomy in malignant ureteral obstruction and concluded that stents as the preferred choice as these are easier to maintain and ureteral stent placement should be recommended whenever feasible. If the malignant obstruction precludes a stent placement, then PCN is a safe alternative.

Dr. Guo and colleagues from China, presented in page 915 (5) a nice systematic review about the prevalence and clinical risk factors in patients diagnosed with incidental prostate cancer (IPC) during certain surgeries (transurethral resection of the prostate [TURP], open prostatectomy [OP], and holmium laser enucleation of the prostate [HoLEP]) after clinically suspected benign prostatic hyperplasia (BPH) and concluded that the prevalence of IPC was similar amongst patients undergoing TURP, HoLEP, and OP for presumed BPH. Interestingly, increased PSA level was the only independent predictor of increasing risk of IPC after BPH surgery rather than age and prostate volume. Hence, future research should focus on predictors which accurately foretell the progression of prostate cancer to determine the optimal treatment for managing patients with IPC after BPH surgery.

Dr. Favorito and colleagues from Brazil performed in page 930 (6) a interesting translational research about the anatomical aspects of the kidney surface in human fetuses during the second gestational trimester and concluded that the number of renal clefts has a great variation, weak correlation and no tendency to decrease during the 2nd gestational trimester. The number of clefts in right kidney of total sample and female fetuses has a significant development with age.

Dr. Carneiro and colleagues from Brazil and USA performed in page 952 (7) an interesting study about the role of remote proctoring during the initial training phases of a robotics curriculum using surgical robot skills simulator exercises and concluded that robotic performance increased significantly over three attempts for simulation exercises of low, medium, but not high-complexity. Proctoring, either in-person or remotely, has a positive impact on approval performance, particularly in intermediate tasks.

Dr. Gonzalez and colleagues from Brazil and Argentina performed in page 961 (8) a nice study about the surgical techniques (open- OP vs laparoscopic –LP - vs robotic -RALP pyeloplasty) to treat ureteropelvic junction obstruction and concluded that minimally invasive surgery for the management of UPJO in children is gaining more acceptance, even in patients younger than 1-year-old. Operative time continues to be significantly shorter in OP than in LP and RALP. Hospital stay was shorter in RALP compared to the other techniques. No differences were found in com- plication rates, type of complications, and reoperation rate.

Dr. Yilmaz-Oral and colleagues from Turkey performed in page 971 (9) a nice experimental study about the possible healing effect of combination treatment with a hydrogen sulfide (H2S) donor, sodium hydrosulfide (NaHS) plus tadalafil on partial bladder outlet obstruction (PBOO)-induced bladder dysfunction and concluded that the combination therapy has beneficial effects on bladder dysfunction via regulating both H2S and nitric oxide pathways as well as downregulation of oxidative stress and hypoxia. The synergistic effect of H2S and nitric oxide is likely to modulate bladder function, which supports the combined therapy for enhancing clinical outcomes in men with BPH/LUTS.

The Editor-in-chief expects everyone to enjoy reading.

## References

[1] Ribeiro A, Bastos JM, de Figueiredo AA, Cândido TC, Guércio WB, Zica BO (2022). Enuresis and upper airway obstruction: BNP and ADH hormones behavior before and after airway surgery. Int Braz J Urol.

[2] Carvalho TA, Vasconcelos MMA, de Bessa J, Bastos JM, Dutra MF, Guimarães ICO, Lima EM, E Silva ACS, Mrad FCC (2022). Relationship between primary monosymptomatic enuresis and process toilet training: a case-control. Int Braz J Urol.

[3] Hu X, Wu Y, Yang P, Wang J, Wang P, Cai J (2022). Performance of 68Ga-labeled prostate-specific membrane antigen ligand positron emission tomography/computed tomography in the diagnosis of primary prostate cancer: a systematic review and meta-analysis. Int Braz J Urol.

[4] Gauhar V, Pirola GM, Scarcella S, De Angelis MV, Giulioni C, Rubilotta E, Gubbiotti M, Lim EJ, Law YXT, Wroclawski ML, Tiong HY, Castellani D (2022). Nephrostomy tube versus double J ureteral stent in patients with malignant ureteric obstruction. A systematic review and meta-analysis of comparative studies. Int Braz J Urol.

[5] Guo Z, He J, Huang L, Wang Z, Hu P, Wang S, Bai Z, Pan J (2022). Prevalence and risk factors of incidental prostate cancer in certain surgeries for benign prostatic hyperplasia: A systematic review and meta-analysis. Int Braz J Urol.

[6] Favorito LA, Lobo MLP, Fernandes AV, Gallo CM, Sampaio FJB (2022). Kidney surface development in human fetuses: study applied to radiological diagnosis. Int Braz J Urol.

[7] Carneiro A, Claros OR, Cha JD, Kayano PP, Apezzato M, Wagner AA, Lemos GC (2022). Can remote assistance for robotic surgery improve surgical performance in simulation training? A prospective clinical trial of urology residents using a simulator in south america. Int Braz J Urol.

[8] González ST, Rosito TE, Tur AB, Ruiz J, Gozalbez R, Maiolo A, Tavares PM, Gorgen ARH, de Kencht EL, Madarriaga YQ, Weller S, Tobia IP, Castellan M, Corbetta JP (2022). Multicenter comparative study of open, laparoscopic, and robotic pyeloplasty in the pediatric population for the treatment of ureteropelvic junction obstruction (UPJO). Int Braz J Urol.

[9] Yilmaz-Oral D, Kaya-Sezginer E, Asker H, Gur S (2022). Co-administration of sodium hydrosulfide and tadalafil modulates hypoxia and oxidative stress on bladder dysfunction in a rat model of bladder outlet obstruction. Int Braz J Urol.

